# Considerations for developing and implementing a safe list for alien taxa

**DOI:** 10.1093/biosci/biad118

**Published:** 2024-02-02

**Authors:** Sabrina Kumschick, Laura Fernandez Winzer, Emily J McCulloch-Jones, Duran Chetty, Jana Fried, Tanushri Govender, Luke J Potgieter, Mokgatla C Rapetsoa, David M Richardson, Julia van Velden, Dewidine Van der Colff, Siyasanga Miza, John R U Wilson

**Affiliations:** Centre for Invasion Biology in the Department of Botany and Zoology at Stellenbosch University, Stellenbosch, South Africa; South African National Biodiversity Institute, Kirstenbosch Research Centre, Cape Town, South Africa; Centre for Invasion Biology in the Department of Botany and Zoology at Stellenbosch University, Stellenbosch, South Africa; South African National Biodiversity Institute, Kirstenbosch Research Centre, Cape Town, South Africa; Centre for Invasion Biology in the Department of Botany and Zoology at Stellenbosch University, Stellenbosch, South Africa; South African National Biodiversity Institute, Kirstenbosch Research Centre, Cape Town, South Africa; South African National Biodiversity Institute, Kirstenbosch Research Centre, Cape Town, South Africa; Department of Horticultural Sciences at Cape Peninsula University of Technology, Cape Town, South Africa; Centre for Agroecology, Water, and Resilience at Coventry University, Coventry, England, United Kingdom; Centre for Invasion Biology in the Department of Botany and Zoology at Stellenbosch University, Stellenbosch, South Africa; South African National Biodiversity Institute, Kirstenbosch Research Centre, Cape Town, South Africa; Centre for Invasion Biology in the Department of Botany and Zoology at Stellenbosch University; Department of Biological Sciences at the University of Toronto-Scarborough, Toronto, Ontario, Canada; South African National Biodiversity Institute, Kirstenbosch Research Centre, Cape Town, South Africa; Centre for Invasion Biology in the Department of Botany and Zoology at Stellenbosch University; Centre for Invasion Biology in the Department of Environmental Sciences, Faculty of Science at Rhodes University, Makhanda, South Africa; Centre for Invasion Biology in the Department of Botany and Zoology at Stellenbosch University; Institute of Botany at the Czech Academy of Sciences, Průhonice, Czech Republic; Centre for Invasion Biology in the Department of Botany and Zoology at Stellenbosch University; Centre for Sustainability Transitions at Stellenbosch University, Stellenbosch, South Africa; Centre for Invasion Biology in the Department of Botany and Zoology at Stellenbosch University, Stellenbosch, South Africa; South African National Biodiversity Institute, Kirstenbosch Research Centre, Cape Town, South Africa; South African National Biodiversity Institute, Kirstenbosch Research Centre, Cape Town, South Africa; Centre for Invasion Biology in the Department of Botany and Zoology at Stellenbosch University, Stellenbosch, South Africa; South African National Biodiversity Institute, Kirstenbosch Research Centre, Cape Town, South Africa

**Keywords:** biological invasions, list approaches, regulation, stakeholders, trade

## Abstract

Many species have been intentionally introduced to new regions for their benefits. Some of these alien species cause damage, others do not (or at least have not yet). There are several approaches to address this problem: prohibit taxa that will cause damage, try to limit damages while preserving benefits, or promote taxa that are safe. In the present article, we unpack the safe list approach, which we define as “a list of taxa alien to the region of interest that are considered of sufficiently low risk of invasion and impact that the taxa can be widely used without concerns of negative impacts.” We discuss the potential use of safe lists in the management of biological invasions; disentangle aspects related to the purpose, development, implementation, and impact of safe lists; and provide guidance for those considering to develop and implement such lists.

Most countries of the world depend heavily on alien taxa for agriculture, forestry, horticulture, the pet trade, and many other uses (Dehnen‐Schmutz et al. 2007, Drew et al. 2010, Lockwood et al. 2019). However, alien taxa can cause significant negative impacts, both directly (e.g., by affecting crop yields or by threatening native species with extinction) and indirectly (e.g., by affecting ecosystem or food system resilience; e.g., Vilà et al. 2010, Weidenhamer and Callaway 2010, Pyšek et al. 2020). The challenge is to assess the benefits alien taxa can provide against the potential negative environmental and socioeconomic impacts of those same taxa. One of the simplest and most pragmatic mechanisms to address this challenge is through the development of lists that act as tools to guide action and policy (García-de-Lomas and Vilà 2015, Pergl et al. 2016). For example, there are lists of taxa that pose a high risk of invasion and impact that should be avoided (i.e., prohibited lists), lists of regulated taxa that can be used under certain conditions, and lists of taxa that present a low risk and are therefore deemed safe to use without restrictions (i.e., approved lists; Young 2006). It is worth noting that all such lists are only useful for taxa intentionally introduced and traded and not for unintentional introductions where other approaches are needed (e.g., pathway-based approaches; Woodford et al. 2016).

Prohibited lists include taxa that are known or predicted to be harmful and that are therefore subject to regulations limiting or prohibiting their importation or use (e.g., Pergl et al. 2016). Note that we include in this term all lists of species that are not allowed, pre- and postborder. Such lists have been a significant focus of research in invasion science (Wilson et al. 2017) and are the focus of globally significant conservation targets. For example, target 6 of the Kunming–Montreal Global Biodiversity Framework specifies the need for efforts aimed at “preventing the introduction and establishment of priority invasive alien species” to mitigate global biodiversity loss. Although prohibited lists might stimulate management action and guide decision-making (e.g., McGeoch et al. 2010, Kumschick and Richardson 2013), they can have unintended consequences. In Australia, for example, strict import bans placed on traded alien reptiles indirectly contributed to numerous unmonitored (illegal) introductions (Stringham et al. 2021). Furthermore, prohibited lists are often reactive rather than proactive, whereby species are listed only once they become invasive and negative impacts have already occurred. The regulation of problematic alien taxa for specific, limited uses is also arguably highly complicated. Criteria that are necessary and sufficient to prevent invasions need to be specified to outline the conditions under which a taxon can be used, and measures to monitor compliance with the conditions might be needed (Datta et al. 2020). As a result, safe lists of approved species can in some circumstances be more effective than prohibited lists (Hulme 2015, de Volder et al. 2020). Similar to prohibited lists, safe lists require scientific evidence and rigorous risk assessment for proposed taxa, but they are relatively straightforward in terms of the set of criteria that are needed. Nonetheless, these approaches still differ in their construction, development, and aims.

The underlying ethos of a safe list is to recognize that there is a demand for particular types of taxa and to provide guidance as to which taxa can be used safely so that this demand can be met without creating damaging invasions. Formally, we define a safe list in the present article as “A list of taxa that are alien to the region of interest and are considered of sufficiently low risk of invasion and impact that the taxa can be widely used without concerns of negative impacts.” We use the term *taxa* rather than *species*, because, although most regulations and lists are based on species, there are cases where it makes more sense to list taxa either below or above the species level. Safe lists recognize the use of alien taxa in addressing specific human needs or wider societal benefits, acknowledging their ongoing use, and providing options for stakeholders.

Safe lists are known by different terms in different industries. For example, in the horticultural industry, they are often referred to as *green lists* or *permitted lists* (Csurhes et al. 2006, Dehnen-Schmutz 2011), but in the pet trade, they are commonly referred to as *positive lists* (table 1; e.g., Toland et al. 2020, Warwick and Steedman 2021). It is important to note that, although safe list approaches seem to be increasingly implemented, the majority have not been published in the scientific literature (e.g., Gardening Responsibly 2021), and the criteria used are generally inconsistent (Toland et al. 2020).

**Table 1.** Examples of safe lists that have been proposed or implemented.

		Term for a safe list			Legally	Only aliens	
Taxon	Industry	used in the reference	Criteria included	Focus region	binding	considered	Reference
Plants	Horticulture	Green list	Y	General, example for Britain	N	Y	Dehnen-Schmutz (2011)
Plants	Horticulture	Green list	Y	Spain	N	N	Bayón and Vilà (2019)
Plants Plants Plants Plants	Horticulture Landscaping Aquatic Trade Horticulture	Green list Green list Green list Low risk list	Y Y Y Y	Great Britain Great Britain Great Britain Australia	N N N N	N N N N	Plant Alert Team (2021a) Plant Alert Team (2021b) Plant Alert Team (2021c) Gardening responsibly (2021)
Plants	Horticulture	Permitted list	Y	Australia	N	N	Csurhes and colleagues (2006)
Plants	Border biosecurity	Permitted list	N	Australia	Y	Y	Spafford Jacob and colleagues (2004)
Plants	Border biosecurity	Permitted list (white list)	N	New Zealand	Y	N	Hulme (2020)
Animals	Pet trade	Positive list	Y	Global (review)	N	NA	Warwick and Steedman (2021)
Mammals	Pet trade	Positive list	Y	Belgium (also mentions the Netherlands)	Y	N	Di Silvestre and van der Hoeven (2016)
Animals	Pet trade	Positive lists (review)	Y	Global (review)	Y	N	Toland and colleagues (2020)
Plants	Border biosecurity and plant trade	White list	N	India	Y	Y	Banerjee and colleagues (2021)
Plants	Bioenergy feedstocks	White list	Y	United States	Y	N	Quinn and colleagues (2015)
NA	NA	White list (clean list)	N	Global (applied in Argentina, Australia, and New Zealand)	Y	Y	Burgiel and Perrault (2011)
Plants	Trade and transport	White list (permitted list)	Y	Australia	Y	N	Invasive species council (2009)
Plants (trees)	Forestry	White lists	Y	Europe	Y	NA	Pötzelsberger and colleagues (2020)
NA	NA	White lists	Y	Global	Y	NA	McNeely and colleagues (2001)
NA	NA	White lists	N	United States (review)	N	N	Simberloff (2006)
NA	NA	White lists (clean list)	N	Global	Y	NA	Burgiel and colleagues (2006)

*Abbreviations:* N, no; NA, not applicable; Y, yes.

Given the potential benefits of safe lists, the increasing frequency and diversity of approaches used, and the uncertainty as to when and how such safe lists can be practically implemented, the approach requires further consideration. In the present article, we propose a roadmap to guide the creation of safe lists—specifically, exploring the purpose, development, implementation, and impact of safe lists—with the aim of supporting policymakers, industry stakeholders, and other decision-makers who might consider using the safe list approach.

## The purpose of safe lists

**Figure 1.**
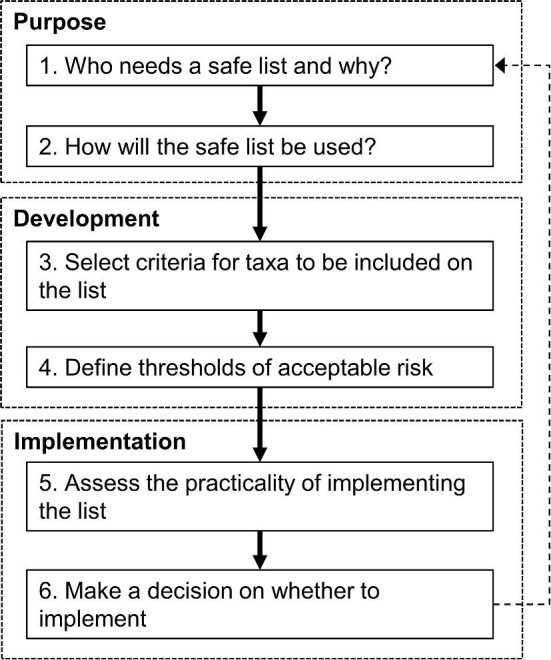
A proposed roadmap to follow when considering a safe list for alien taxa. The dashed arrow highlights that the process is iterative.

At the outset it is important to be clear about the purpose of any proposed safe list, specifically: ‘Who needs a safe list and why?’ and ‘How will the safe list be used?’ (figure 1).

### 1. Who needs a safe list and why? 

A wide variety of stakeholders could make use of a safe list of alien taxa, tailored to the specific needs of the related industry or purpose. Stakeholders may include policymakers, conservation organizations, land managers, and traders in specific industries (e.g., ornamental horticulture and the pet trade). Table 1 shows a diversity of safe lists that have been developed or implemented for different industries and regions.

The appropriateness of a safe list must be considered in the same context as any other tool designed to assist with managing biological invasions. This is further discussed below (i.e., whether the safe list approach should be implemented), but first, it is important to consider whether a safe list approach is feasible at all. Safe lists generally adopt a taxon-based approach (cf. McGeoch et al. 2016). In practice, this means that it must be possible for those using the safe list to be able to differentiate taxa and in particular to distinguish alien taxa from native taxa (e.g., Essl et al. 2018) and within alien taxa those that are considered safe. In some instances, however, identifying individual taxa can be difficult, and so pathway- or site-based approaches might be more appropriate. For example, rather than considering the risks of moving specific aquatic organisms in ballast water or in interbasin water transfers, it is more practical to focus on the pathway itself—that is, on the risks of invasions of moving water per se (Woodford et al. 2016). For such unintentional introductions, a safe list is not a suitable tool.

If a safe list looks like a possible option, it is then important to set the scene. Is the list to include particular taxonomic or functional groups only—for example, Cactaceae or large mammals? Should the list be specific to particular uses or for specific industries or sectors, such as forestry, horticulture, or the pet trade? At what spatial scale and for which region is the safe list to be applied? The spatial scale will define the climatic zones and biomes for which the taxa must be considered safe. Different scenarios could be set out following each step outlined in the proposed roadmap for developing a safe list (figure 1).

### 2. How will the safe list be used?

**Figure 2.**
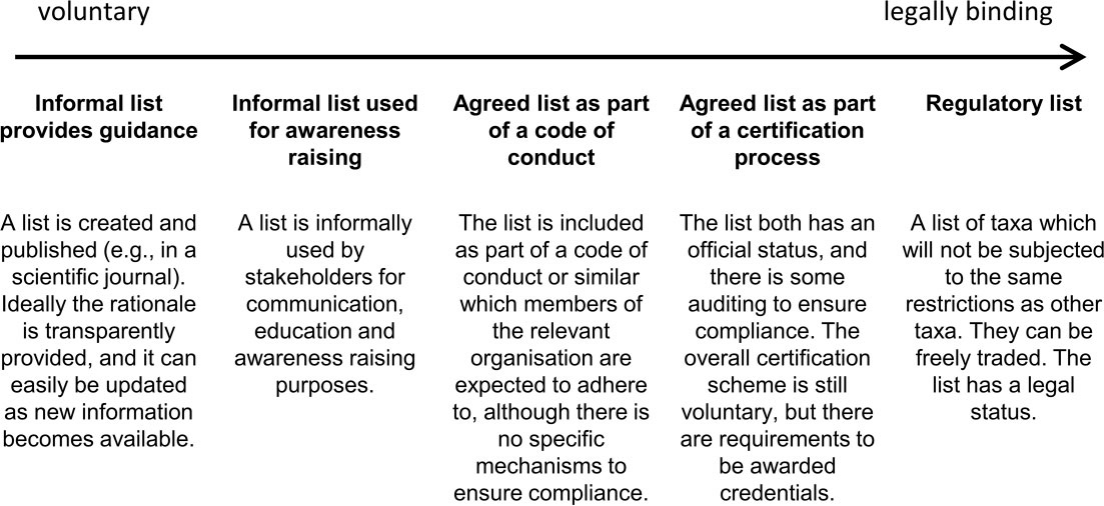
Different options for how legally binding the safe list can be for the industry or sector for which it is produced. The arrow shows a gradient from voluntary to legally binding. As the lists become more legally binding, greater effort is expected to make the list accessible, changes to the list will tend to require greater consultation and formal processes, and the effort to implement the list will increase (to the point where there is some legal enforcement).

Besides considering the scope and context of a safe list, it is also important to clearly define how the list will be used and how its potential implementation aligns with other interventions, including legislation. We identified some demonstrative scenarios for how safe lists could be used, recognizing that these scenarios are archetypes and do not provide an exhaustive overview of all possible scenarios (figure 2).

At one end of the continuum, safe lists can simply be informative tools providing guidance and recommendations. In many cases, they fall into this category when developed as academic exercises. However, for a safe list to have a substantial impact and be widely adopted, it is essential for it to be clearly owned by those who are directly affected by its implementation. Without such ownership, it is unlikely to lead to significant uptake or impact. Following on from that, a list may be formulated specifically to gain recognition within the respective industry or other relevant bodies of interest. Such voluntary lists can be used to create awareness about biological invasions and come without legal repercussions for anyone involved in trade. Examples of informal and voluntary lists are included in table 1.

As a slight modification of the previous approach, the list can also be implemented as part of a code of conduct by a stakeholder body or a group of interested and affected parties (e.g., Heywood and Sharrock^ 2013^). These are typically codeveloped by the affected industry in collaboration with scientists (e.g., Brundu et al. 2020 for forestry). Although the adoption of a code of conduct is voluntary, such codes acknowledge an issue and signal a commitment to address it. Codes of conduct require a strong relationship between scientists, nongovernmental organizations, and industry to promote compliance and uptake (Field et al. 2013) and ideally, also involve government agencies in their development. However, because there is no enforcement nor repercussions for actions that contravene the code, voluntary agreements and codes of conduct often prove effective only if there are incentives for their use (Hulme et al. 2018).

If it is deemed crucial for safe lists to be adhered to, a mechanism to enforce compliance might be necessary. This will usually require both monitoring and some form of accreditation. In this approach, the activities of stakeholders are audited and certified, typically by an independent body. Only those complying with the code of conduct (and any safe list contained within it) are allowed to use specific marketing or branding (see the supplemental material for examples), whereas those not honoring the code of conduct are sanctioned (e.g., through negative marketing, reduced sales, or other economic penalties). A list of certified traders could be made publicly available to increase consumer support for legitimate and environmentally responsible traders. Such a response in consumer behavior may in turn improve compliance with traders (Ward and Phillips 2008). There could be various levels of uptake and implementation. Certain traders perhaps only stock safe list taxa (e.g., a nursery selling only native or safe list alien plants). Alternatively, there could be a label or a section in the respective industries, similar to an organic or a “water wise” label (e.g., Kelly 2015). Of course, certification schemes present their own set of challenges: They can create concerns around the appropriateness of hiring a gatekeeper, the need to ensure processes are transparent and fair, and that no perverse incentives are created. Such self-regulation also crucially depends on trust between and within industry, the public, and government. Addressing biological invasions is often a public good problem, and so regulation might be warranted for safe lists to be effective.

The final scenario we present is one where a governmental organization has ownership of a safe list and enforces compliance. International legal frameworks offer guidance on how such an approach could work. For example, the World Trade Organization, which regulates global trade under its “Agreement on the Application of Sanitary and Phytosanitary Measures,” allows the restriction of trade in a species based on a risk assessment (FAO 2013). Safe lists could therefore be regulated as a permitted list of taxa that can be traded or imported without restrictions (Burgiel and Perrault 2011). In such a case, only taxa on a safe list could be freely imported or traded, whereas all other taxa would require a comprehensive risk assessment—a guilty-until-proven-innocent approach (cf. Hulme et al. 2018). Although this approach may be effective in managing biological invasions, it is restrictive and can be regarded as draconian or unnecessarily punitive leading to conflicts of interest and, potentially, a higher prevalence of illegal markets (e.g., Stringham et al. 2021). An unintended consequence of safe lists could therefore be increased illegal trade, which is coupled with a myriad of other social, environmental, and governance problems (e.g., Cardoso et al. 2021, Fukushima et al. 2021, but see Di Silvestre and van der Hoeven 2016). Furthermore, amending regulations and policies can be time consuming and cumbersome, and so a regulatory safe list might be expected to be less dynamic than a guiding document or broad codes of conduct.

In the end, which scenario is implemented depends on the stakeholders involved. For example, a safe list cannot be legally binding if government is not involved. Similarly, nongovernmental groups working alone may not have much leverage with industries, but the threat from government of a legally binding list could push industries to develop their own voluntary safe lists.

## Development

Regardless of whether the safe list is compulsory or voluntary, it is important to consider how such a list is developed and publicized. As for prohibited lists, the development of a safe list should be informed by a scientific, independent, evidence-based, and transparent process (Simberloff 2003, Dehnen-Schmutz 2011). Furthermore, if the list is to be adopted by the relevant stakeholders, suitable engagement and awareness campaigns are important (Shackleton et al. 2019), similar, for example, to existing programs for sustainable resource use (Field et al. 2013, Hulme et al. 2018).

### 3. Select criteria for taxa to be included on the list

Essentially, the taxa of interest need to be subjected to a risk assessment and be deemed low risk to be acceptable for a safe list. Many risk assessment protocols have been developed for biological invasions (e.g., Kumschick and Richardson 2013), however, most of them focus on the identification of high risk taxa for prohibited lists. Ideally, there should be a mechanistic understanding of what prevents each taxon on the safe list from becoming invasive and having negative impacts (e.g., Bufford and Daehler 2014). However, this level of autecological detail is lacking for most taxa. Instead, safe lists are routinely based on criteria that are believed to be directly related to risk, but that are in some cases proxies—for example, using the number of nurseries that sell a particular taxon to determine propagule pressure (Dehnen-Schmutz 2011).

The most appropriate criteria will depend on the intended use of the safe list. However, there are some frequently used criteria (table 2). One of the most widely applied criteria is invasiveness elsewhere or invasion history (e.g., Dehnen-Schmutz 2011, Toland et al. 2020, Warwick and Steedman 2021). Species distribution modeling or climate matching is used to supplement such information and identify taxa for which the current or future environments are suitable for an invasion to occur.

**Table 2.** Criteria considered by different sources to develop safe lists in the horticulture and pet trades.

Criterion	Rationale	Issues	Source
Current invasion or legal status	If a taxon is flagged as invasive or is regulated in the target region, then it should not be included on a safe list	This either depends on the robustness of a separate regulatory process or on the assumption that invasiveness per se is proof of sufficient impact or impact potential.	Bayón and Vilà (2019), Gardening Responsibly (2021)
Invasiveness elsewhere	One of the more robust predictors of invasiveness is “invasiveness elsewhere”. If the taxon is flagged as invasive in other regions, or present in any national checklist of invasive species, it should not be considered as safe	There can be biotic and abiotic limits to invasion such that a taxon is safe in one context but not in another (e.g., biotic resistance will vary depending on native taxa present)	Dehnen-Schmutz (2011), Bayón and Vilà (2019), Toland and colleagues (2020), Gardening Responsibly (2021), Warwick and Steedman (2021)
Established or naturalized elsewhere	This criterion assesses whether the taxon has become established or naturalized (self-replacing, reproducing outside of captivity or cultivation) anywhere in the world where it is not native. Some safe-listing protocols directly exclude taxa if they are established anywhere	Establishment does not imply rapid spread or impact and depends on many biotic and abiotic factors that might not be present at all sites, and so although it can be concerning, it might exclude many taxa that do not pose a significant risk	Bayón and Vilà (2019)
Invasiveness of closely related taxa	This criterion questions whether there are invasive taxa in the same genus (or very closely related) in a similar climate or in the target region. This requires the study of phylogenetic trees to identify closely related taxa, on the basis of the assumption that they could share invasive traits	There are very few genera where all taxa are invasive, and the traits relevant to invasiveness and impact may not be phylogenetically conserved	Gardening Responsibly (2021)
Climatic or habitat matching	If the target region has a similar climate or habitat to the native or invaded areas worldwide, then it might be expected that establishment is possible. Such analyses usually involve the matching of environmental conditions (often focused on climate variables).	Alien taxa often occupy different climatic and habitat niches in introduced areas to native ranges, which can lead to incorrect predictions. A suitable climate and habitat are necessary but not sufficient conditions for an invasion.	Bayón and Vilà (2019), Gardening Responsibly (2021)
Response to climate change	Taxa that currently cannot establish or spread because of climatic barriers can give the impression to be safe, but future changes in climate could alter this scenario. Therefore, the potential for changes in response to climate change needs to be a factor in safe listing. For example, species distribution modeling under several climate change scenarios could be considered before including a species on a safe list	There is a need to set a temporal window (e.g., by 2050) and chose realistic scenarios, recognizing that these are projections under set circumstances. In any case, it is important to give an expiry date on a safe list	Dehnen-Schmutz (2011), Gardening Responsibly (2021)
Residence time	The longer a taxon has been present without showing any tendencies to become established, the safer it is, i.e., the taxon has had many opportunities to invade but has not done so. Consequently, for a taxon to be considered safe, it must have been present in the target area for a sufficiently long time and not found outside cultivation/captivity	Many taxa do not have a long history as aliens, and so this criterion can restrict many taxa. Moreover, residence time is only a proxy for opportunities to invade, because taxa might have been introduced to unsuitable sites or in low numbers or there can be a limiting factor that, once lifted, will allow an invasion to occur (i.e., a lag phase *sensu stricto*).	Dehnen-Schmutz (2011), Gardening Responsibly (2021)
Introduction and propagule pressure	Higher propagule pressure is often associated with higher invasion success. Therefore, if a taxon has a high propagule pressure (i.e., many opportunities to invade) but has not caused an invasion or impact, it is assumed that the taxon is safe.	The issues are similar to those for residence time. Moreover, actual propagule pressure is often unknown and difficult or impossible to determine retrospectively. Various proxies are often used [e.g., marketing time, frequency (proportion of shops selling a taxon), planting frequency in a sample of gardens, volume, and prices] that might be less related to opportunities to invade.	Dehnen-Schmutz (2011)
Dispersal pathways	This criterion considers the dispersal capabilities of the taxon, and whether it can be dispersed by other abiotic or biotic means. The easier a taxon can disperse, the more likely it is to spread and become invasive	Human-mediated dispersal can be difficult to quantify. The relative importance of dispersal traits and pathways differs according to the land use (e.g., urban versus rural)	Gardening Responsibly (2021)
Type of reproduction	Taxa able to reproduce and spread asexually have fewer reproductive restrictions than taxa that only reproduce sexually, and a single individual can form a new population	Sexual reproduction favors increased dispersal potential. Some taxa that reproduce sexually (e.g., pines) are very successful invaders. Furthermore, genetic diversity through sexual reproduction can favor establishment and invasion success	Gardening Responsibly (2021)
Fecundity	Highly fecund taxa are able to produce higher numbers of offspring, therefore increasing the propagule pressure. This can include the number of seeds produced, germination rate and seed viability, dormancy periods, and rate of reproduction	Site-specific characteristics can still act as barriers to establishment, although such characteristics might also change over time (e.g., with climate change, presence of other species)	Gardening Responsibly (2021)
Age of reproductive maturity	The younger a species can reproduce, the higher the chances of establishing self-sustaining populations earlier. For example, for plants, does the species produce viable seed within the first 3 years (for a herbaceous species) to 5 years (for a woody species) after germination?	Site-specific characteristics can still act as barriers to establishment. This criterion might affect how quickly an invasion begins, but not ultimately whether a damaging invasion will occur or not.	Gardening Responsibly (2021)
Environmental and socioeconomic impacts	If the taxon causes impact elsewhere or is predicted to cause impact on the basis of which impact mechanisms are likely to occur, it is likely to cause impacts in the target region (cf. invasiveness elsewhere and of close relatives)	Data on impacts are often lacking and difficult to obtain or quantify. Socioeconomic and environmental impacts are often context-specific and not necessarily generic, although evaluations of impact mechanisms can improve the specificity of predictions.	Bayón and Vilà (2019), Gardening Responsibly (2021), Warwick and Steedman (2021)
Public health and safety	The taxon should not pose an unacceptable risk to human health (zoonotic diseases, injuries, poisoning, etc.)	These effects can depend on the context in which the taxon occurs (e.g., surrounding human population density and demographics, land-use type, sanitation standards). For a specific activity using the taxon there might not be a marginal increase in risk when compared with using other taxa (i.e., the activity is inherently risky rather than the taxon *per se*). For example, keeping ungulates on game farms might increase the density of ticks, but does the alien taxon proposed for the safe list lead to more ticks, additional species of tick, or higher transmission of disease?	Toland and colleagues (2020), Gardening Responsibly (2021), Warwick and Steedman (2021)

Another set of criteria considers the potential negative impacts, including whether there have been impacts elsewhere (Bayón and Vilà 2019, Gardening Responsibly 2021). Impacts can be related to the environment (cf. EICAT; IUCN 2020, Volery et al. 2020) or to human well-being and livelihoods (cf. SEICAT; Bacher et al. 2018). In both cases, if detrimental impacts have been observed in one region, it can generally be assumed, in the absence of contrary evidence, that similar harmful impacts may occur in another (see also Kumschick et al. 2024).

A lack of invasiveness and impact elsewhere does not, however, indicate that a taxon is safe. Only a small proportion of all taxa worldwide has been introduced outside of their native ranges, but a smaller fraction has been given appropriate opportunities (e.g., been planted in a suitable area over a long period of time) to become invasive and have an impact. Therefore, many safe list schemes focus on criteria that provide evidence that a taxon has had opportunities to become invasive and cause harmful impacts but has not done so, specifically considering how long a taxon has been in the region (residence time) and the frequency and number of individuals that have been introduced into the environment (propagule pressure; table 2). However, this information is limited for many taxa in many regions. Such lack of information can greatly inhibit the process of identifying taxa suitable for a safe list (e.g., Dehnen-Schmutz 2011).

### 4. Define thresholds of acceptable risk

A safe list approach requires the development of thresholds that determine the level of risk above or below which a taxon can be considered safe. In the decision-making process, there is often a choice to be made between granting opportunities for societal or commercial interests and minimizing the potential for biological invasions and their impacts. The concepts of false positives (i.e., listing a taxon with unacceptable impacts as safe) and of false negatives (i.e., of limiting a taxon without sufficient impacts) are useful in this context (cf. supplemental figure S1). Different scenarios require different levels of acceptance of the two outcomes. By selecting more informative criteria, the suitable option space is increased. To minimize the number of invasive or harmful species in the trade, one would aim to keep the number of false positives as low as possible. However, this could lead to some safe species classified as high risk, which could negatively affect the revenue of the industry as less species would be considered safe. Despite this potential negative effect, because of the implications of a species being added on the safe list (and therefore its promotion and likely increase in trade), false positives should be avoided. Research could improve our knowledge or confidence, allowing for better decisions regarding the appropriate size of the list. Thresholds will need to be determined for acceptable levels of risk in relation to the context and region in question and for all criteria used to inform the development of the lists (Wonham et al. 2013, Bayón and Vilà 2019). Importantly, if there is a systematic error in one of the criteria used to construct a safe list, then multiple taxa might be categorized as safe for the same incorrect reason leading to multiple damaging invasions (supplemental figure S1).

Substantial uncertainty exists in how a taxon may respond to a new environment, and in some situations, developing a list that appropriately and adequately balances risks and benefits will be impossible. Some activities might, therefore, simply be incompatible with a safe list approach. However, safe lists can be improved by selecting the most informative criteria and by conducting specific research on the taxa or industry in question to ensure the availability of new and updated information for more taxa concerning these criteria. Such improvements can act to reduce the potential for errors (in terms of false negatives and false positives).

## Implementation

Finally it is important to consider how a safe list will be implemented. Is it practical? And is it the best (or at least a good) option? (Figure 1).

### 5. Assess the practicality of implementing the list

Various practical issues will affect how feasible it is to construct and implement a safe list. First, evidence for criteria pertaining to risk relies greatly on the evaluation of past introductions of alien taxa (Richardson et al. 2011). As a result, only taxa that are well known, well studied, and widely introduced are likely to be included on a safe list, and understudied taxa may not be appropriately considered.

Ideally, all taxa present in a certain industry should be assessed for inclusion in a safe list using the selected criteria. However, the reality of large numbers of taxa being traded can be prohibitive of such an approach, and some preselection for further assessment (for example in stakeholder workshops) might have to be made to reduce the initial workload.

Importantly, given the possibility of false negatives and especially of false positives, safe lists need to be updated when needed, and species reassessed according to an agreed timeframe. This requires administration and agreement regarding the processes to be followed. For legally binding lists, administration is usually coordinated by government agencies, but for other types of lists, responsibility for this role needs to be determined to ensure effective implementation.

As with any attempt to create a list on the basis of taxa, it is important to recognize variation in nomenclature and how this can change over time (Isaac et al. 2004), including revisions, reclassification, and misidentifications (Regan et al. 2002, McGeoch et al. 2012). Native and invasive species have been confused in some cases because of nomenclatural or taxonomic errors, with adverse effects on management (Geller 1999, Pyšek et al. 2013). Similarly, misidentification can lead to difficulties around the use and trade of alien taxa. In particular, the use of common names is prominent in trade across different industries (e.g., Keller and Lodge 2007). This can lead to uncertainties regarding which species are used or available in trade and therefore creates difficulties when evaluating taxa for inclusion on a safe list. Furthermore, subspecific variation or hybridization may lead to other taxonomic issues that affect the development and implementation of a safe list (see the supplemental material).

The process of creating a safe list should include considerations of the temporal validity of the list. Specifying a shelf life and determining cutoff dates for revision (e.g., every 3 years) are necessary. Furthermore, processes for adding new taxa and removing listed taxa should be stipulated (Ochoa-Ochoa et al. 2019). These processes require careful consideration as removing taxa from the list (i.e., declaring them as no longer safe) may cause conflict and bring into question the validity or value of the list. Safe lists need to be static enough to be trusted by stakeholders but dynamic enough to ensure that changing conditions and new information are accommodated timeously.

The spatial area over which the safe list applies should be considered and specified and, when legally binding, should ideally apply to the entire region to which the legislative framework applies. However, in some cases, the criteria might be applied at finer spatial scales—for example, at a state or provincial level or for specific biomes or protected areas. Where different climatic zones or biomes provide varied opportunities for invasion within a singular region (van Wilgen et al. 2020), the taxa should be considered safe in all environments within the region to qualify for listing. This is particularly important because there are generally limited capabilities and insufficient barriers to restrict the movement of individuals (and taxa) within countries (Nelufule et al. 2022). Similarly, although all the environments within a country might be regarded as unsuitable for a specific alien taxon, and the taxon is therefore considered safe at a national level, neighboring countries can still be at risk of invasion (e.g., Faulkner et al. 2020). Furthermore, it is important to note that the possible effects of climate change could result in changes regarding environment suitability for different taxa (see the supplemental material; Hulme 2017). Therefore, the threat of interregional spread and potential conflicts between respective legal frameworks needs to be considered when compiling safe lists (Maher et al. 2023).

### 6. Make a decision on whether to implement

As a final step, we propose revisiting one of the initial considerations: determining whether a safe list is the most suitable tool to suit the scenario or whether there are alternative options that might be preferable. This includes going back to assessing different options if the initial purpose for a safe list does not seem feasible, useful, or effective (figure 1).

Possible effects of a safe list on human well-being are closely linked to how it is implemented. If safe lists were to be implemented as fully legally binding instruments with only safe listed taxa available in trade (figure 2), the variety of taxa legally available to consumers could be drastically reduced. This is particularly challenging for some taxonomic groups. For example, parrots are highly popular in trade but cause major problems in many parts of the world where they have been introduced (Souviron-Priego et al. 2018), and a long-entrenched preference for certain species exists (Mori et al. 2017). Consequently, it has proven difficult to enforce regulations in the avian pet trade (Souviron-Priego et al. 2018), because species that are problematic remain popular in trade, and there may often be insufficient information to promote alternative species to buyers. In the ornamental horticultural sector, the range of plants available to buyers was shown to be an important determining factor for consumers when deciding where to buy their plants, after the quality and costs of plants (Dunn et al. 2020). Therefore, legally enforced safe lists could drastically influence the recreational benefits that society enjoys within their homes and the environment.

Consumer choice is also affected by several characteristics that might not automatically be represented in a safe list. For plants, key traits that affect consumer’s selection include flower size and number, foliage, color, hardy varieties, and plant vigor (Reichard and White 2001, Hulme et al. 2018, van Kleunen et al. 2018). For the reptile trade, the popularity of traded species was linked with traits such as size, colors and patterns, and whether the species was dangerous (van Wilgen et al. 2010). In the ornamental crayfish trade, small taxa from lentic habitats are preferred (Cucholl and Wendler^ 2017^). In most cases, these traits are not linked to the risks the species pose with regards to their invasiveness and impacts. This complicates both the task of compiling a safe list and providing guidelines on suitable alternatives to consumers. If consumer demands are not met, noncompliance is likely. A lack of choice could also negatively affect consumer behavior. Psychological research has found that having more choices can improve individual satisfaction, because people prefer making their own decisions rather than having them dictated externally (Botti and Iyengar 2006). Furthermore, demand can be driven by societal trends (e.g., Siriwat et al. 2020), which might not be met by the taxa on a safe list.

An important implication of safe lists, particularly if they are legally binding, is that the livelihoods of traders whose primary commodity is excluded from the safe list might be threatened. This is of especial concern as some marginalized people can depend on the trade of alien plants or animals to make a living (e.g., Shackleton et al. 2011). The potential for such situations must be identified and treated sensitively. However, the converse can also be true: Safe lists may facilitate improved livelihoods by making the trade and use of certain alien taxa socially or legally acceptable, thereby creating market confidence. Some development projects, for example, promote the cultivation of alien plants for various purposes, such as biofuels (Blanchard et al. 2011). Developing safe lists for such projects could benefit the communities in several ways, because they would ensure the taxa recommended do not lead to unintended negative consequences down the line.

A recent study showed that, when consumers were provided with the knowledge of the positive ecological impacts of using selected native plants in their gardens, they were less interested in alien alternatives (Anderson et al. 2021). The creation of public knowledge is therefore a powerful tool to curb biological invasions (Hulme 2017), and safe lists could provide a more positive environment for incentivization for noninvasive or environmentally friendly choices in the market. Suppliers and consumers are also more motivated to reduce ecological harm when they are provided with direction (Beaury et al. 2021, Gabellini and Scaramuzzi 2022).

In some contexts, traders understand the negative impacts of biological invasions, prefer not to be associated with causing invasions, and are therefore supportive of measures to regulate species (e.g., Cronin et al. 2017). However, traders require clarity as to which taxa can be traded so as to avoid investing in stock and the promotion of taxa that might later be found to be undesirable or declared illegal. Traders within various industries are likely to have differing levels of enthusiasm for market change or motivation to comply with safe lists, with the extent of buy-in often being dependent on their knowledge about which taxa are alien, which are invasive, and any corresponding legal and financial implications (Humair et al. 2014).

Within the public sector, the success of a safe list hinges on uptake, which is influenced by formal pressures such as policy and legislation and informal pressures such as societal norms (e.g., influence between individuals or groups to follow specific trends or concepts; Tenge et al. 2004). The process, however, is contingent on the flow of knowledge. Stakeholders need to buy into the safe options, and a safe list should provide viable alternatives to popular (but harmful) taxa. Participative and consultative processes will therefore be vital to ensure public uptake, both from traders and consumers. The use of eco labeling, pamphlets, and posters with easy to understand and informative infographics around the positive implications of safe listed taxa may assist in creating awareness (e.g., Plant Alert Team 2021a).

The cost of development and enforcement of a safe list will likely increase along with increasing legality, as will the complexity of the process required to add or remove taxa from the list, reducing how dynamic and responsive the lists can be over time. Most concerning is the potential that industry stakeholders and consumers cannot be held liable if safe listed taxa become invasive should the list be implemented as a legally binding safe list (figure 2).

Finally, the process should be iterative. Protocols and work flows need to be developed to accommodate new information, noting that any alterations to the list (in particular the removal of taxa previously considered “safe”) may cause significant confusion, create conflict for traders and consumers, and bring into question the validity and reliability of the list. The nature of the changes allowed and the requirements for making changes need careful consideration, noting that a safe list must have a clear review process, including assigned review responsibilities and an expiry date.

## Conclusions

Conservation messages are often negative (“doom and gloom”); but positive stories can be much more powerful in stimulating action. Invasion science and international targets related to biological invasions focus mostly on identifying and prioritizing harmful and potentially harmful alien taxa. In contrast, safe lists represent a proactive and positive approach to managing biological invasions and can guide policy development. Instead of focusing on prohibition of harmful and potentially harmful alien taxa, safe lists highlight taxa that can be used without causing negative impacts. They are an aid to stimulate more sustainable practices and support positive messaging for greater engagement in conservation efforts.

Safe lists allow for a context-specific approach to managing biological invasions. The taxa included in a safe list can and need to be tailored to the specific ecological context of a region. If these lists are robustly developed and thoughtfully implemented, then safe lists can effectively mitigate the risks of biological invasions because—by promoting the use of low-risk taxa—safe lists can prevent the introduction and spread of potentially harmful taxa. However, although there are globally numerous examples of prohibited lists (e.g., McGeoch et al. 2012), there are still relatively few examples of safe lists (e.g., Dehnen-Schmutz 2011). This begs the question, which taxa can be used for safe lists?

In this article, we have outlined the essential steps to be followed when contemplating the development of a safe list (figure 1). Importantly, the development and implementation of safe lists aligns with international agreements that require risk assessments for the regulation of trade and the introduction of alien species. This process enables transparency and the promotion of trust among stakeholders and the public. This transparency can enhance the acceptance and implementation of safe lists. Although there is a need for further research into the traits that confer invasiveness and the impacts of invasive species, and for research that can therefore inform future policy development and refinement of safe lists, we believe the roadmap presented in the present article provides guidance on developing and implementing safe lists in a cautionary, context-specific, and appropriate manner.

## Supplementary Material

biad118_Supplemental_File

## Author Biography

Sabrina Kumschick (sabrina.kumschick@gmail.com), Laura Fernandez Winzer, Emily J. McCulloch-Jones, Tanushri Govender, Dewidine Van der Colff, and John R. U. Wilson are affiliated with the Centre for Invasion Biology in the Department of Botany and Zoology at Stellenbosch University, in Stellenbosch, South Africa and with the South African National Biodiversity Institute, Kirstenbosch Research Centre, in Cape Town, South Africa. Duran Chetty is affiliated with the South African National Biodiversity Institute, Kirstenbosch Research Centre, in Cape Town, South Africa, and with the Department of Horticultural Sciences at Cape Peninsula University of Technology, in Cape Town, South Africa. Jana Fried is affiliated with the Centre for Agroecology, Water, and Resilience at Coventry University, in Coventry, England, in the United Kingdom. Luke J. Potgieter is affiliated with the Centre for Invasion Biology in the Department of Botany and Zoology at Stellenbosch University and with the Department of Biological Sciences at the University of Toronto-Scarborough, in Toronto, Ontario, Canada. Mokgatla C. Rapetsoa is affiliated with the South African National Biodiversity Institute, Kirstenbosch Research Centre, in Cape Town, South Africa, the Centre for Invasion Biology in the Department of Botany and Zoology at Stellenbosch University and with the Centre for Invasion Biology, in the Department of Environmental Sciences, Faculty of Science, at Rhodes University, in Makhanda, South Africa. David M. Richardson is affiliated with Centre for Invasion Biology in the Department of Botany and Zoology at Stellenbosch University and with the Institute of Botany at the Czech Academy of Sciences, in Průhonice, Czech Republic. Julia van Velden is affiliated with the Centre for Invasion Biology in the Department of Botany and Zoology at Stellenbosch University and with the Centre for Sustainability Transitions at Stellenbosch University, in Stellenbosch, South Africa. Siyasanga Miza is affiliated with the South African National Biodiversity Institute, Kirstenbosch Research Centre, in Cape Town, South Africa.
