# Supplementary material for: Considerations for developing and implementing a safe list for alien taxa
Source: Bioscience. 2024 Feb 2;74(2):97–108. doi: 10.1093/biosci/biad118 (PMC10880065; doi:10.1093/biosci/biad118)
Supplement: biad118_Supplemental_File [file biad118_supplemental_file.docx]

# Supplementary Material

## Methods

The underlying themes in this paper were developed during two workshops. Firstly, during a workshop in June 2022, a Strengths, Weaknesses, Opportunities and Threats (SWOT) analysis of safe lists was performed by most of the authors, following a 4-step approach:

*Step 1: Defining the scope of the analysis*
The different aspects of the SWOT analysis were explained by the facilitator (SK), i.e., strengths and weaknesses including internal factors, and opportunities and threats alluding to external factors (see also Scolozzi et al. 2014). It was decided that the analysis should have a global focus, cover all taxonomic groups, and span any potential industry that could apply a safe listing approach for alien taxa. Only intentionally traded organisms were included in the exercise.

*Step 2: Key factors for each SWOT component*
All participants noted down at least one factor for each of the four components of the SWOT analysis on different coloured post-it papers, each separated into Strengths, Weaknesses, Opportunities and Threats.

*Step 3: Organising according to themes*The participants were divided into four groups, one for each component of the SWOT analysis, to organise the notes according to themes. The themes were then presented to the whole group. Some notes were moved to other more relevant SWOT components, and clarified by the original participant who took the note, if further explanation was required. All notes and identified themes were collated.

*Step 4: Identification of overarching themes*
In a second workshop, some of the authors (SK, LFW and EMJ) grouped all notes and comments into main themes regardless of whether they were listed as a strength, weakness, opportunity or threat, and merged some of the topics identified.

# Additional considerations regarding the development and implementation of a safe list

***Response to climate change***

Climate change can affect biological invasions in at least three ways. Firstly, extreme climatic events could lead to increased dispersal of propagules via wind and water (Pryor et al. 2012). Secondly, changes in surface temperatures and general climatic conditions may render previously unsuitable environments suitable for colonisation (Hulme et al. 2017). Thirdly, climate change can favour invasive species through increased fecundity or resistance to pathogens and pesticides, therefore hindering management efforts (Rahel and Olden 2008, Diez et al. 2012). Climate change is thus a factor that may influence the longevity and validity of a safe list, bringing into question the long-term ‘safety’ of a species as it may change the behaviour of alien and invasive species in the future. Therefore, measures need to be adopted to mitigate the possible effects of climate change on the safe list.

***Hybridisation***

Hybridisation can be a threat for the implementation of a safe list and can result in several possible genetic changes (Landry et al. 2007) leading to fitness gains or losses (Charlesworth and Willis 2009). Those types of changes can directly influence species’ competition and thus invasion success. Intraspecific hybridisation is possible amongst cultivars or breeds of the same species, and this can lead to invasiveness (Ellstrand and Schierenbeck 2000, Wolfe et al. 2007). For example, safe cultivars could revert to their invasive forms if they hybridise with other cultivars or with their wild type congeners. However, it should be noted that not all instances of intraspecific hybridisation result in increased invasiveness (Ellstrand and Schierenbeck 2000, Wolfe et al. 2007) and that all the right genetic combinations that complement the new environment where invasive traits are selected must be met (Culley and Hardiman 2009). Therefore, it is crucial that the implications of intraspecific hybridisation are thoroughly evaluated before considering a taxon for inclusion on a safe list.

***Cultivars and varieties***

Plant cultivars can vary significantly from the parent species, which may present as different functional traits in the environmental niches that they occupy (Wilson and Mecca 2003, Wilson et al. 2004, Knox and Wilson 2006). This can also apply to different breeds of animals (Galukande et al. 2013, Leroy et al. 2020). Such differences could translate into unique invasion risks (Gordon et al. 2016, Datta et al. 2020). Most frameworks to assess risk are designed for species level assessments (however, see Gardening Responsibly 2022). Additionally, multiple introductions of different subspecies or cultivars are likely to be a stimulus for intraspecific hybridisation. This can also be a threat for the implementation of a safe list, as hybridisation can result in several possible genetic changes (Landry et al. 2007) leading to fitness gains or losses (Charlesworth and Willis 2009). This might seem intractable, but knowledge of how different lineages behave (e.g., their propensity for hybridisation) can provide some useful insights as to whether these are concerning issues.

**
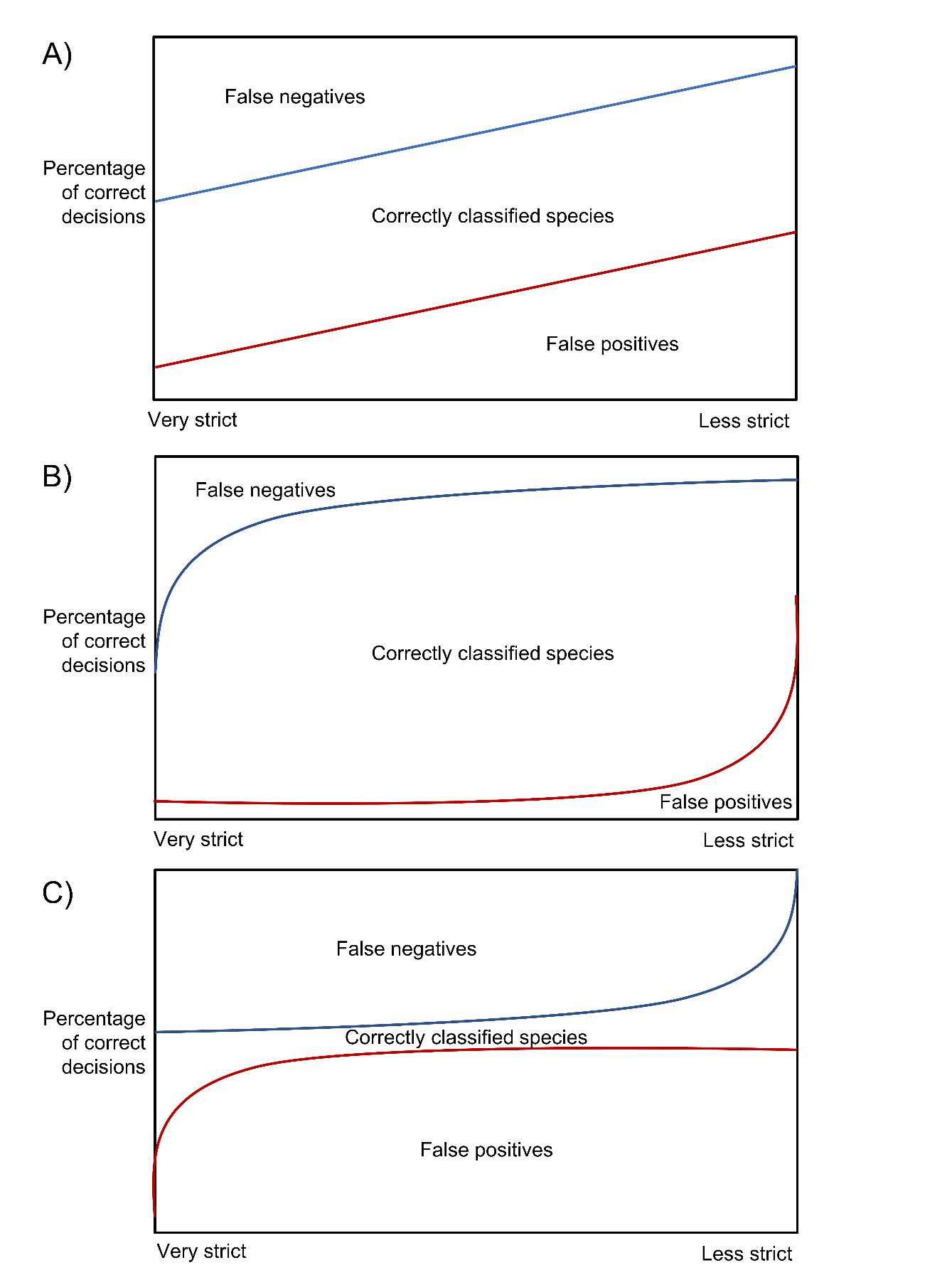
**

## Figure S1. Any assessment of risk needs to consider the trade-off between false positives and false negatives. The figures depict hypothetical percentages of false negatives, correctly classified, and false positive species assuming the same x number of species are assessed under varying cut-off levels for criteria from very strict to less strict. False negatives are species classified as high risk (i.e., species excluded from the safe list) even though they are safe; false positives are species classified as safe which are in fact high risk. Arguably false positives are of more concern that false negatives. In the context of safe lists, false positives can lead to damaging invasions that have massive impacts in the long-term with the costs borne by society as a whole, while the cost of false negatives is more in terms of reducing the opportunities available to particular citizens. One complication that is widely recognised in biological invasions, is that the costs of false negatives is immediate and often occurs to a specific group of people who might be very vocal in the restrictions placed on their rights and so significant political pressure can be exerted to make decisions that will increase the risk of invasions. Selection criteria (particularly correlates of harm) often require taxa to have had substantial opportunities to cause harm, and so many taxa that have not already had such opportunities would be excluded (a more robust mechanistic case would be needed to justify the inclusion of such taxa on a safe list). This figure depicts the effect the selection of criteria and cut-off levels can have on the likelihood of false negatives and false positives across three different scenarios: (A) An archetypical representation of the problem, however the error rates are likely to be neither a linear function of list length nor symmetrical. (B) An ideal situation for a safe list, where for a certain length list there are very few false negatives or positives. (C) In other cases, there might always be significant errors, indicating that a safe list is likely an inappropriate tool.

**Supplementary Materials References**

Charlesworth B. 2009. Effective population size and patterns of molecular evolution and variation. Nature Reviews Genetics 10:195–205.

Culley TM, Hardiman NA. 2009. The role of intraspecific hybridization in the evolution of invasiveness: a case study of the ornamental pear tree *Pyrus calleryan*a. Biological Invasions 11: 1107-19.

Datta A, Kumschick S, Geerts S, Wilson JRU. 2020. Identifying safe cultivars of invasive plants: six questions for risk assessment, management, and communication. NeoBiota 62: 81–97.

Diez JM et al. 2012. Will extreme climatic events facilitate biological invasions?. Frontiers in Ecology and the Environment 10: 249–57.

Ellstrand NC, Schierenbeck KA. 2000. Hybridization as a stimulus for the evolution of invasiveness in plants?. Proceedings of the National Academy of Sciences 97: 7043–50.

Galukande E, Mulindwa H, Wurzinger M, Roschinsky R, Mwai AO, Sölkner J. 2013. Cross-breeding cattle for milk production in the tropics: achievements, challenges and opportunities. Animal Genetic Resources 52: 111–125.

Gordon DR et al. 2016. Weed risk assessments are an effective component of invasion risk management. Invasive Plant Science and Management 9: 81-3.

Knox GW, Wilson SB. 2006. Evaluating north and south Florida landscape performance and fruiting of ten cultivars and a wild-type selection of *Nandina domestica*, a potentially invasive shrub. Journal of Environmental Horticulture 24: 137-42.

Landry CR, Hartl DL, Ranz JM. 2007. Genome clashes in hybrids: insights from gene expression. Heredity 99: 483-93.

Leroy G, Boettcher P, Besbes B, Peña CR, Jaffrezic F, Baumung R. 2020. Food securers or invasive aliens? Trends and consequences of non-native livestock introgression in developing countries. Global food security 26: 100420.

Pryor SC, Barthelmie RJ, Clausen NE, Drews M, MacKellar N, Kjellström E. 2012. Analyses of possible changes in intense and extreme wind speeds over northern Europe under climate change scenarios. Climate dynamics 38: 189-208.

Rahel FJ, Olden JD, 2008. Assessing the Effects of Climate Change on Aquatic Invasive Species. Conservation Biology 22: 521–533.

Scolozzi R, Schirpke U, Morri E, D'Amato D, Santolini R. 2014. Ecosystem services-based SWOT analysis of protected areas for conservation strategies. Journal of environmental management 146: 543-51.

Wilson SB, Mecca LK. 2003. Seed production and germination of eight cultivars and the wild type of *Ruellia tweediana*: A potentially invasive ornamental. Journal of Environmental Horticulture 21: 137-43.

Wilson SB, Mecca LK, Gersony JA, Thetford M, Raymer JS. Evaluation of 14 butterfly bush taxa grown in western and southern Florida: II. 2004. Seed production and germination. HortTechnology 14: 612-8.

Wolfe LM, Blair AC, Penna BM. 2007. Does intraspecific hybridization contribute to the evolution of invasiveness?: an experimental test. Biological Invasions 9: 515-21.
